# A Functional Magnetic Resonance Imaging Study to Investigate the Utility of a Picture Imagination Task in Investigating Neural Responses in Patients with Chronic Musculoskeletal Pain to Daily Physical Activity Photographs

**DOI:** 10.1371/journal.pone.0141133

**Published:** 2015-10-23

**Authors:** Ann M Taylor, Ashley D Harris, Alice Varnava, Rhiannon Phillips, Justin O. Taylor, Owen Hughes, Antony R Wilkes, Judith E Hall, Richard G Wise

**Affiliations:** 1 Institute of Infection and Immunity, Cardiff University, Wales, United Kingdom; 2 Russell H. Morgan Department of Radiology and Radiological Science, The Johns Hopkins University, Baltimore, United States of America; 3 F. M. Kirby Center for Functional Brain Imaging, Kennedy Krieger Institute, Baltimore, United States of America; 4 Department of Psychology, Swansea University, Singleton Park, Swansea, Wales, United Kingdom; 5 School of Psychology, Cardiff University, Wales, United Kingdom; 6 Institute of Primary Care and Public Health, Cardiff University, Wales, United Kingdom; 7 Barry Chiropractic Clinic, Barry, Wales, United Kingdom; 8 Bronllys Pain and Fatigue Management Centre, Brecon, Powys, Wales, United Kingdom; 9 Cardiff University Brain Research Imaging Centre (CUBRIC), School of Psychology, Cardiff University, Wales, United Kingdom; University of Vienna, AUSTRIA

## Abstract

Pain-related anxiety and fear are associated with increased difficulties in attention, increased awareness of pain, impaired disengagement from pain, and can moderate the effects of attentional coping attempts. Accurately assessing the direct impact of pain-related anxiety and fear on pain behavior has proved difficult. Studies have demonstrated no or limited influence of pain-related fear and anxiety on behavior but this may be due to inherent problems with the scales used. Neuroimaging has improved the understanding of neural processes underlying the factors that influence pain perception. This study aimed to establish if a Picture and Imagination Task (PIT), largely developed from the Photographs of Daily Activity (PHODA) assessment tool, could help explore how people living with chronic pain process information about daily activities. Blood oxygenation level dependent (BOLD) functional magnetic resonance imaging (fMRI) was used to compare brain responses in patients with chronic musculoskeletal pain (CMSKP) (n = 15) and healthy controls (n = 15). Subjects were asked to imagine how they would feel mentally and physically if asked to perform daily activities illustrated in PIT. The results found that a number of regions involved in pain processing saw increased BOLD activation in patients compared with controls when undertaking the task and included the insula, anterior cingulate cortex, thalamus and inferior and superior parietal cortices. Similarly, increased BOLD responses in patients compared to controls in the frontal pole, paracingulate and the supplementary motor cortex may be suggestive of a memory component to the responses The amygdala, orbitofrontal cortex, substantia nigra/ventral tegmentum, putamen, thalamus, pallidum, inferior parietal (supramarginal and angular gyrus) and cingulate cortex were also seen to have greater differences in BOLD signal changes in patients compared with controls and many of these regions are also associated with general phobic responses. Therefore, we suggest that PIT is a useful task to explore pain- and movement-related anxiety and fear in fMRI studies. Regions in the Default Mode Network remained active or were less deactivated during the PIT task in patients with CMSKP compared to healthy controls supporting the contention that the DMN is abnormal in patients with CMSKP.

## Introduction

The way in which chronic musculoskeletal pain (CMSKP) is perceived is modified by many factors [1–7]; the strongest and most consistent being pain-related fear, anxiety, and catastrophizing [1]. Pain-related anxiety and fear are associated with difficulty in attention, increased awareness of pain, impaired disengagement from pain, and can moderate the effects of attentional coping attempts [2, 8–14]. Thibodeau et al [15] found that fear of injury contributes to pain-related anxiety and functional impairment causing catastrophic interpretation of pain. Such is the impact of pain-related anxiety, merely the intention to perform a painful movement can induce behavioral defensive responses [16, 17]. Reduction in movement-related fear and anxiety predict improvements in functioning, reduced affective distress, pain, and interference with daily activity [18–20], and therefore understanding these processes has important therapeutic implications.

In 2000, Vlaeyen and Linton [21] introduced their Fear-Avoidance (FA) model; a model which described how pain disability, affective distress, and physical disuse develop as a result of persistent avoidance behaviors motivated by pain- and movement- related fear. High fear of pain can lead to hypervigilance for both pain and pain-related information [21–23]. Cognitions shape not only psychological outcomes such as emotional functioning but also the nervous system activity underlying pain perception [24, 25]. Therefore, it is unsurprising that maladaptive pain cognitions are associated with emotional and behavioral responses leading to activity avoidance, disability, depression [23, 26–29] and predicts future pain [30]. Burton et al [31] recommend that good quality trials should be performed on how providing information and education can reduce pain-related fear avoidance beliefs and improve coping in the prevention of low back pain. However, Pincus et al [32] felt this statement was premature as a better understanding is needed of the relationship between beliefs about pain, movement, pain- related fear and avoidance and behavior.

A number of studies have examined the impact of pain- and movement- related fear, anxiety and avoidant behavior on CMSKP patients undertaking physical activities aiming to investigate the association between movement-related fear and anxiety and behavioral performance [33–43]. Participants with high fear of pain and (re)injury undertook less physical activity [34, 35, 37, 39], had higher activity induced pain [36], more functional limitations [37] and undertook less leisure-time activity [34]. However, a number of studies did not find an association between kinesiophobia (fear of movement) and physical activity [33, 38, 40–43].

While pain-related fear and anxiety have important roles in understanding and managing chronic pain, accurately assessing their direct impact on pain behavior has proved difficult. Studies have used a range of physical capacity tasks that have demonstrated no or limited influence of pain-related fear on behavior [40, 43–45]. There are several things that may account for these findings: lack of sensitivity in the scales used to record pain- and movement- related fear, undisclosed or unrecognized fears, painful movements not defined in the questionnaires, and/or the physical capacity task chosen may not to tap into an individual’s specific fears [32]. To improve understanding of the processes underlying fear and anxiety in chronic pain, individual information about movements that are perceived to cause fear and anxiety should be obtained, relating this specifically to the individuals involved in the research [32].

Neuroimaging has improved the understanding of neural processes underlying the factors that influence pain perception [46–48]. Pain-processing is modified by experience and psychological factors thought to amplify pain signals, such as pain- and movement- related fear, catastrophizing and anxiety. These may lead to changes in central neural mechanisms leading to central sensitization and a chronic hyperalgesic state [49–51]. Past experiences influencing movement-related fear are reliant on memory and recall [52, 53] and a number of neuroimaging studies have investigated this by asking healthy volunteers to imagine pain [54–56]. A number of common brain regions were activated including: ACC, thalamus, insula, prefrontal and parietal cortices. Consistent amygdala and insula hyperactivity has been shown in a range of anxiety disorders and in fear conditioning in healthy subjects, which is interpreted to reflect exaggerated engagement of fear circuitry [57]. However, there is evidence to suggest that naturally occurring pain differs greatly from experimentally induced pain in relation to anxiety and fear avoidance [58]. The subjective meaning of pain in healthy participants may be very different from those with CMSKP [2, 13] and this may also be true for the neural mechanisms underlying movement-related anxiety and fear where little is currently known in patients with CMSKP. Furthermore, previous research has highlighted that as chronic pain develops several biological changes occur, including up-regulation of centres that process pain and down-regulation of endogenous anti-nociceptive mechanisms [59]. Therefore, studies need to be undertaken in chronic pain populations rather than extrapolating findings from healthy volunteers to this group.

Shimo et al [60] examined pain-related fear in a group of chronic low back pain patients. They hypothesized that visualization of a painful event may trigger painful memories, thus provoking the affective dimension of pain. They investigated neural correlates of affect processing in subjects with and without LBP using a picture of a man carrying luggage in a half-crouching position. All subjects with LBP reported experiencing discomfort and some reported experiencing pain. In contrast to subjects without LBP, subjects with LBP displayed activation of the cortical areas related to pain and emotion including the insular cortex, supplementary motor area and pre-motor area, cerebellum, thalamus, pulvinar, posterior cingulate cortex, hippocampus, fusiform gyrus and angular gyrus. This suggests that the virtual LBP stimuli caused memory retrieval of unpleasant experiences and the authors concluded that this may be associated with prolonged chronic LBP conditions.

Barke et al [61] investigated the neural correlates of fear of movement in two groups of women who had CLBP (high and low avoidance) and compared fear responses to those with arachnophobia and healthy controls. High avoidance pain patients did not show increased activation (compared with low fear avoidant participants or to neutral pictures) in areas presumed to respond to phobias and fear [57]. These findings contrasted with the activations in ‘fear regions’ seen when avoidant patients viewed general fear-related pictures or when arachnophobic viewed pictures of spiders. As a result, the authors concluded that the results did not support the fear component of the Fear Avoidance Model. Barke et al [61] proposed a number of explanations for their findings. These explanations were all well argued however, there was no resolution offered as to why there were no neuronal fear responses to Photographs of Daily Activities (PHODA) questionnaire.

The study was designed to investigate how people living with CMSKP process information about daily activities by using an individually tailored Picture and Imagination Task (PIT) derived from the Photographs of Daily Activity Assessment Tool (PHODA) [62]. From the back pain literature discussed above, there are still inconsistencies in the evidence base and further work to be undertaken. Therefore, functional magnetic resonance imaging (fMRI) was used to identify regions where there are differences in blood oxygenation level dependent (BOLD) responses in CMSKP patients compared to controls while completing the PIT task. We hypothesized that there will be increased BOLD activation in patients compared to controls in regions associated with pain-related fear and anxiety in response to images of potentially painful movements, in the absence of experimentally induced nociceptive stimulation.

## Material and Methods

### Participants

Following Dyfed Powys Ethics Committee approval, participants who provided informed written consent were recruited for the study. Fifteen patients were recruited from a pain management program and a multidisciplinary pain clinic in South Wales. Fifteen age and sex matched healthy controls were recruited from the Cardiff University School of Psychology’s volunteer panel. Criteria for patient inclusion in the study were: a physician-diagnosis of chronic non- malignant pain (International Association for the Study of Pain; [63]); non-inflammatory musculoskeletal pain; a pain score of 50 or above on a numerical rating scale (NRS) of 0–100 (‘No Pain’—‘Worst Possible Pain’) for average pain experienced over the month prior to enrolment; that lying down would not provoke pain, and participants perceived that they would be comfortable in the scanner. Patients had to have been on stable analgesic regimens for 3 months prior to scanning with no new analgesia or treatment being initiated during this period. It was deemed unethical to stop analgesia prior to scanning. Controls were asked about current pain and included if they had very mild pain, it was not recurrent or persistent, they had no diagnosed pain condition and that any pain was not musculoskeletal in origin. Exclusion criteria for all participants were: metabolic, rheumatoid, vascular, neurological or diagnosed psychiatric disorders, including major depression; the inability to give informed consent, and contraindications to MR scanning.

### Questionnaires and assessment

In an interview conducted 2–4 weeks prior to scanning, patients were asked about their current medication, intensity of pain, and depression, kinesiophobia, catastrophizing and anxiety scores were obtained. All interviews were conducted by the same researcher (AT). Using a numerical rating scale (NRS), where 0 = no pain and 100 = the worst possible pain, patients were asked to indicate the number that best described the worst pain, least pain and average pain intensity over the previous week, average pain intensity over the previous three months and the degree to which the pain interfered with activities of daily living.

Depressed mood was assessed using the Beck Depression Inventory (BDI) [64]. The BDI comprises 21 questions, each item ranging from 0 to 3 points, maximum score 63 points; with scores indicating: <10 no depression, 10–18.7 mild depression, 18.7–25.4 moderate depression, and > 25.4 severe depression [64].

The Tampa Scale of Kinesiophobia (TSK) [65, 66] was used to evaluate fear of movement. The TSK questionnaire comprises 17 items assessing subjective ratings of kinesiophobia. Each item has a 4-point Likert scale with scoring alternatives ranging from ‘strongly disagree’ to ‘strongly agree’. A total score is calculated, ranging from 17 to 68. A greater TSK score indicates a greater degree of kinesiophobia and a cut-off of 37 is typically used to define a high degree of kinesiophobia [23].

Catastrophizing was assessed using the catastrophizing subscale from the Coping Strategies Questionnaire (CSQ-CAT) [67]. The CSQ-CAT includes six items that are scored on a 7-point scale, relating to negative evaluation, helplessness, and catastrophic thoughts about pain. Clinically relevant catastrophizing is defined by a CSQ-CAT score of 11 or above [68].

### PIT development

The activity pictures were taken from a previously validated tool to assess kinesiophobia in low back pain populations [62], PHODA. PHODA was originally developed as a diagnostic tool to determine the perceived harmfulness of different physical activities and movement [62] using photographs of 8 possible movements set against 4 areas of daily occupations and converted into recognizable and frequent activities instead of in terms of their biomechanics. Leeuw et al [69] developed a shortened electronic version of the PHODA (the PHODA-SeV) where patients are requested to drag each photograph along a ‘harmfulness thermometer’ ranging from 0, not harmful, to 100, extremely harmful. The authors contended that the test-retest reliability of the PHODA-SeV over a 2-week time-interval was excellent [69] and found that the construct validity of the PHODA-SeV was supported by consistent relationships with self-report measures of fear of movement/(re)injury, pain, catastrophizing, functional disability, and current pain intensity.

However, the tool itself was not appropriate for the purpose of this study as it requested participants to rate perceived harmfulness of an activity; we did not want to bias the neural responses by suggesting the activities were harmful. To ensure the PHODA pictures were salient for the patient participants, a pilot study had been previously performed using 20 patients with chronic osteoarthritis musculoskeletal pain, age and gender matched with 20 pain-free controls outside the scanner. Participants were asked to imagine how much pain and anxiety they would feel if they were asked to complete the activity represented in the photograph and rate both on a scale of 0 to 3 (0 = ‘no pain’ to 3 = ‘severe pain’, and 0 –‘no anxiety’ to 3 ‘severe anxiety’, respectively). Those activity pictures that were rated the highest for both anxiety and pain in the patient group were the ones that were used in the present study. No patient or matched control from the pilot was invited to participate in the present study.

Six categories of activity pictures were viewed by the participants and these included exercise, twisting, bending, pull/push, carrying and lifting. Participants viewed 40 activity-related pictures. The seventh category was of 20 resting pictures. Thirty activity pictures were common to all subjects and consisted of 5 photographs from each activity category. During informed consent (between 2–4 weeks before scanning), patients were asked about the type of activity that was most troublesome for them to perform and also how best they rested. These activities were matched to photographs from PHODA and became the ‘bespoke’ pictures (mentioned in Table 1). Ten activity pictures were included in the bespoke activity category and 10 resting pictures were included to reflect how best they rested. This was performed to ensured salience of the pictures. All patients viewed the same photographs as their matched controls.

**Table 1:** Average anxiety and pain ratings.

	Anxiety rated in the Scanner		Pain rated post scanning	
Function	Median (25^th^, 75^th^ quartiles) 0 = no anxiety – 3 severe anxiety	*p* value (Mann-Whitney)	Median (25^th^, 75^th^ quartiles) 0 = no pain – 3 severe pain	*p* value (Mann-Whitney)
Bending	Patients 2 (1, 3)	< .001	Patients 2 (1.6, 2.8)	< .001
	Controls 0 (0, 0)		Controls 0 (0, 0)	
Twisting	Patients 2 (1.4, 2.4)	< .001	Patients 2 (1.4, 2.4)	< .001
	Controls 0 (0, 0)		Controls 0 (0, 0)	
Carrying	Patients 2 (1.8, 3)	< .001	Patients 2 (2, 3)	< .001
	Controls 0 (0, 0)		Controls 0 (0, 0)	
Exercise	Patients 3 (2.2, 2.8)	< .001	Patients 2 (2.2, 2.8)	< .001
	Controls 0 (0, 0)		Controls 0 (0, 0.6)	
Push/pull	Patients 2 (2, 2.8)	< .001	Patients 2 (2, 2.8)	< .001
	Controls 0 (0, 0)		Controls 0 (0, 0.2)	
Lifting	Patients 2 (2, 2.8)	< .001	Patients 2 (2, 2.8)	< .001
	Controls 0 (0, 0)		Controls 0 (0, 0)	
Bespoke	Patients 2 (1.8, 2.6)	< .001	Patients 2 (2, 2.6)	< .001
	Controls 0 (0, 0)		Controls 0 (0, 0.1)	
Resting	Patients 1 (0, 1)	< .001	Patients 1 (0.4, 1.2)	< .001
	Controls 0 (0, 0)		Controls 0 (0, 0)	

### Imaging paradigm for the picture and imagination task

The PIT study used an event-related fMRI design. Subjects were presented with individual pictures of daily activities (e.g. a person lifting a shopping bag, bending to pick something off the floor) and asked to imagine performing the activity. Participants were given these instructions ‘A photograph will be presented for 3 seconds. Study the photograph carefully and imagine that after scanning we will ask you to attempt this activity. Think about how this would make you feel. Imagine how you would feel both mentally and physically during your attempt.’

**Fig 1:**
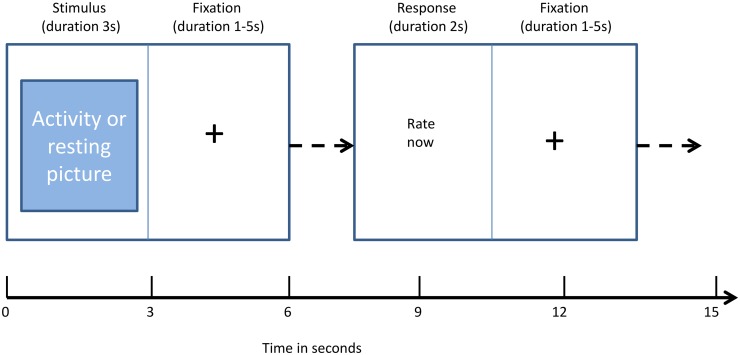
Trial timing. Each trial in the task lasted 7–15 s and was composed of 4 different screens; a photo from either PHODA or a resting activity, a fixation cross, a screen to indicate the subject should respond and ended with a second fixation cross.

Participants were asked to rate how anxious they would be in undertaking the activity shown after the picture was displayed within the scanner (see Fig 1) using a 0 to 3 scale where 0 was no anxiety and 3 was severe anxiety. Participants rated their anxiety using two button response boxes, one held in each hand. Participants used their middle and index finger of their left hand for no anxiety and mild anxiety, respectively, and the index and middle finger of their right hand for moderate and severe anxiety, respectively. The patient group viewed the same photographs as their matched controls, over the same length of time and all were given the same instructions in the scanner. Reference to pain was not made immediately prior to or during scanning to ensure that participants were imagining the activity and were not prompted to imagine pain. It was only after scanning that patients were asked to rate the perceived pain on undertaking the activity using a similar scale to that used in rating anxiety.

Each trial (see Fig 1) lasted between 7s and 15s and consisted of a picture presented on screen for a fixed duration of 3s, a fixation cross for a random number of seconds between 1s and 5s (mean 3s), a response period (‘RATE NOW’) whereby the subject had a fixed 2s to rate their anxiety, and finally another fixation cross for between 1s and 5s (mean 3s) before the commencement of the next trial. The durations of both fixations were in a random fixed order and counterbalanced across the 60 photographs. The whole presentation lasted 11 minutes.

### Participant training

Prior to scanning, subjects completed a practice version of the task in a mock scanner lasting approximately 90s and consisting of 10 trials. All pictures used in the practice session were different to those presented in the scanning session. Thus, subjects practiced the task components of button pressing but were not exposed to the pictures used in the scanner. Responses were reviewed to ensure that the subject understood the task. Participants were asked if they felt comfortable in the mock scanner and were happy to proceed to the main scanner.

### Imaging

Imaging was performed on a 3 T MRI system (HDx, General Electric Healthcare, Waukesha, Wisconsin, USA) using an 8-channel receive-only head coil. E-Prime was used to deliver the functional stimuli. Functional MRI data were acquired with a gradient-echo, echo-planar imaging sequence, scanning parameters were: repetition time (TR)/echo time (TE) = 3000 ms/35 ms, 20.5 cm field of view, acquired on a 64 x 64 matrix with 53 contiguous 3.2 mm slices. Each run consisted of 236 volumes. For anatomic localization, a T1-weighted, three-dimensional fast-spoiled gradient echo acquisition was performed, with a voxel resolution 1x1x1 mm^3^ (scanning parameters included: TR/TE = 7.8/3 ms, 450 ms inversion time) for each participant.

### Image analysis

Analysis of BOLD fMRI data was performed using FSL (FMRIB's Software Library, www.fmrib.ox.ac.uk/fsl). Functional data for each subject was motion corrected (MCFLIRT) [70] and field distortions were corrected (PRELUDE+FUGUE) [71, 72]. Data were smoothed spatially using a 5mm FWHM Gaussian kernel and highpass filtered with a temporal cut off of 100s. At the voxel level, pre-whitening was performed to remove autocorrelations (FILM-FMRIB's Improved Linear Model) [73]. BOLD images were linearly registered to each subject’s structural scan (FLIRT, 6 degrees of freedom) [70, 74] and subsequently registered to standard space (FLIRT, 12 degrees of freedom, followed by FNIRT with warp resolution 10mm)[75].

A general linear model was applied to analyze functional data (individual subject level). The first level analysis included three regressors; (1) the time of display of the picture, (2) the time of the response, (3) the presentation of the picture modulated by the demeaned anxiety response scores reported when viewing the picture. Each of these regressors was convolved with gamma-variate haemodynamic response function.

A second level mixed effects analysis (FLAME) [76] was performed to compare patient and control groups and included BDI as a covariate. In a supplementary second level analysis, an average for each participant’s anxiety score responses rated during the picture viewing task, catastrophizing scores and the TSK scores were included as regressors (demeaned across the group), again including each participant’s BDI as a covariate, demeaned across the group. These regressions were ‘picture + anxiety scores’, ‘picture + TSK scores’ and ‘picture and catastrophizing scores’ looking for a correlation between high scores and areas of BOLD activity. All statistical images were thresholded using clusters determined by a Z>2.3 followed by cluster correction at a significance threshold of *p* = 0.05 [77].

## Results

### Demographics and questionnaires

Thirty participants were scanned (5 male, 10 female for both patients and controls), age range 25 to 83 years old, including 15 patients with pain and 15 age-matched controls. Two patients were left handed and these were also matched to left handed controls. No differences were found between groups in marital status, years in school or number of dependents. Thirteen patients had previously undergone a diagnostic MRI scan and 9 volunteers had previously been scanned as participants in previous studies or for non-pain related clinical reasons. Pain scores, demographic data and psychological variables were compared between groups. Patients’ clinical characteristics are described in Table 2.

**Table 2:** Clinical characteristics of pain patients.

Patient	Age	*Pain sites	Duration of pain (years)
1	29	Knees	2
2	59	Back, neck	1
3	65	Shoulders, hips	3
4	25	Knees, hips	1
5	60	Back, knees	3
6	61	Back, feet	4
7	83	Major joints	20
8	76	Major joints	5
9	65	Major joints	25
10	71	Back, shoulders	25
11	62	Back, shoulders	1
12	38	Back	10
13	64	Major joints	10
14	56	Back and neck	5
15	55	Back	15

*All patients had osteoarthritis.

Patients and controls differed in pain scores and psychological variables (Table 3).

**Table 3:** Psychological variables and pain scores.

Variable	Patient	Control	*p* value
Beck Depression Inventory (<10 no depression, 10–18.7 mild, 18.7–25.4 moderate, > 35.4 severe depression)	23 (12–34)	3 (0–5)	< 0.001
CSQ Catastrophize (0 = Never catastrophize, 36 = Always catastrophize about pain)	14 (3–23)	2 (0–3)	< 0.001
Tampa Scale of Kinesiophobia (The total score ranges between 17 and 68. A high value on the TSK indicates a high degree of kinesiophobia, a score of 37 differentiates between high and low scores)	23 (17–32)	9 (4–14)	< 0.001
Current pain (present pain) (NRS 0–100)	60 (47–68)	0 (0–0)	< 0.001
Worst pain (worst pain imaginable during week) (NRS 0–100)	90 (73–95)	0 (0–0)	< 0.001
Least pain (during week) (NRS 0–100)	35 (25–52)	0 (0–0)	< 0.001
Pain intensity (during week) (NRS 0–100)	64 (50–70)	0 (0–0)	< 0.001
Pain distress (during week) (NRS 0–100)	70 (60–78)	0 (0–0)	< 0.001
Disturbance (during week) (NRS 0–100)	61 (51–83)	0 (0–0)	< 0.001

Median score presented (interquartile range shown in parentheses). Differences between groups were tested using Mann-Whitney tests.

All patients were receiving opioids and all had received a course of physiotherapy, 9 had received education on self-management, but no patient had attended a pain management program or an expert patient program.

The anxiety ratings performed on the button box provided statistically significant differences (*p*< 0.001) between patients and controls for all picture types (Table 1) illustrating that the pictures caused medium to high anxiety in patients but not in controls. Similarly the perceived pain ratings, rated after the scanning session, provided statistically significant differences (*p*< 0.001) between patients and controls for all activity pictures illustrating that patients perceived that the activities would result in moderate to severe pain.

### Imaging

Differences in BOLD responses were seen with patients showing greater activation compared to controls (patients > controls) when viewing the pictures, but not for the other two regressors representing the ‘rate now’ instruction and the button press response. There were no BOLD response differences in which controls showed greater activation compared to patients (controls > patients).

**Fig 2:**
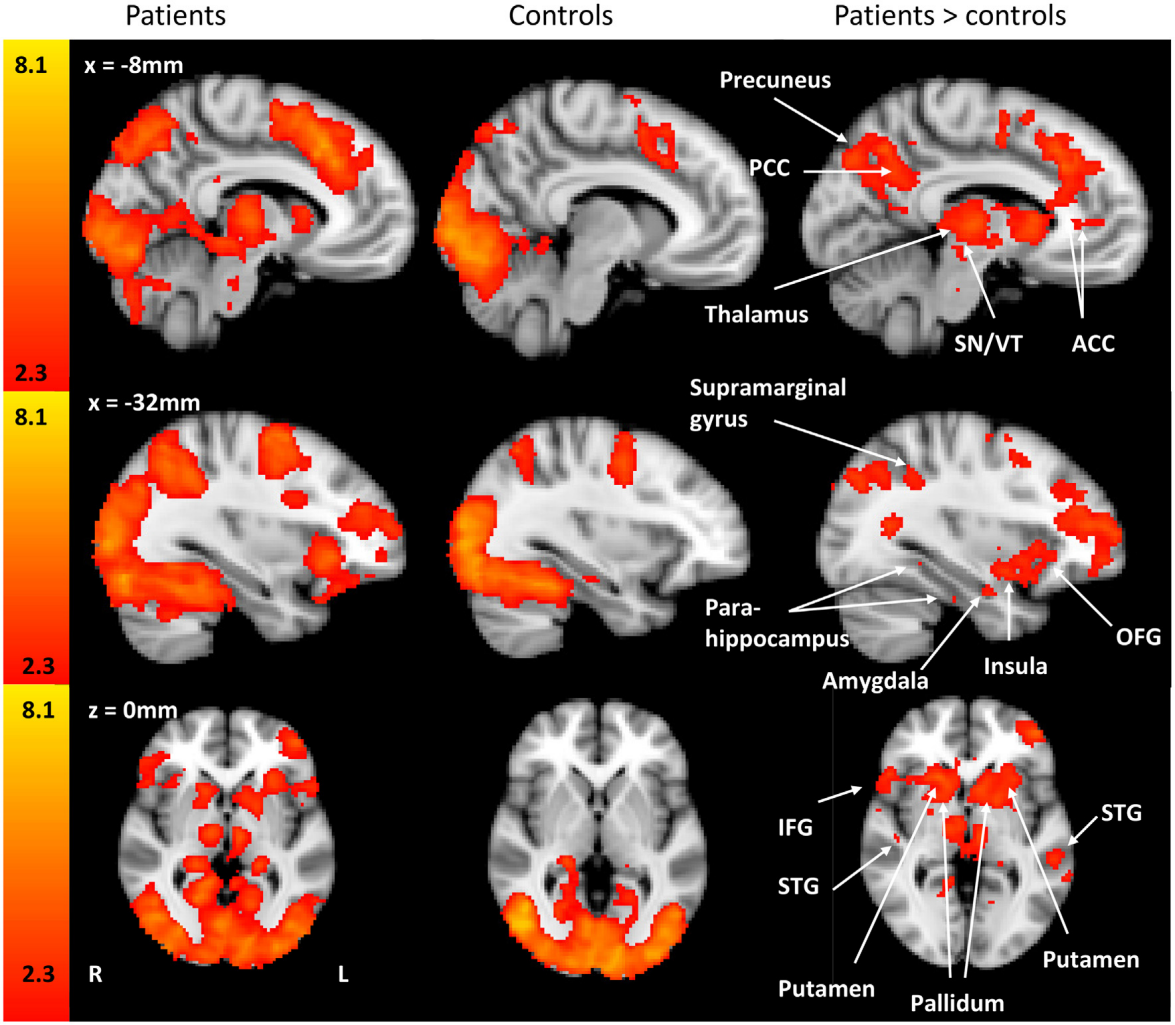
Maps illustrating activation of brain regions during the PIT task activity. Statistical maps of the patient group, the control group and patient > control comparing activation during the PIT task. Patients with CMSKP have significantly different BOLD activation in regions known to be involved in pain processing, phobia and fear conditioning. Each z-statistic map represents these group differences in a whole brain analysis. The color bar shows the scale of the Z-statistic (2.3–8.1). Slice location is identified in white on the figures and presented in millimeters. The z-stat comes from the corrected clusters, the z-stats themselves are not corrected. PCC: posterior cingulate cortex, ACC: anterior cingulate cortex, STG: superior temporal gyrus, OFG: orbitofrontal gyrus, SN/VT: substantia nigra/ventral tegmental region, IFG: inferior frontal gyrus.

**Fig 3:**
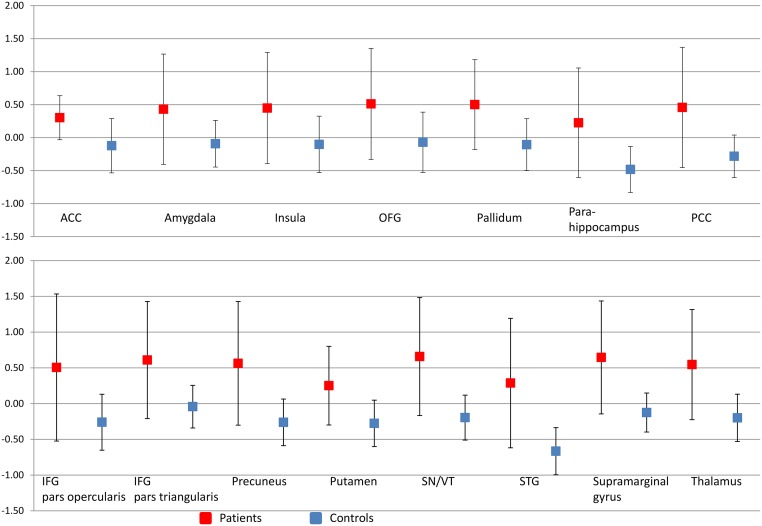
Graphs illustrating the percentage signal change in areas illustrated in Fig 2. The graphs show the percentage signal change with the error bars representing standard deviations across subjects. The percent signal change is calculated across the significantly activated voxels within the Harvard-Oxford atlas defined anatomical region. Images illustrated in Fig 2 are a combination of anatomical and functional data and the graphs represent the direction and magnitude of the signal change within the regions illustrated in Fig 2 Because these activations are already defined as significant, no further statistical tests were performed.

In the initial second level analysis (no inclusion of kinesiophobia, catastrophizing or anxiety, inclusion of demeaned BDI), BOLD signal differences during viewing of the pictures showed greater activation in patients compared to controls (patients>controls) in a number of bilateral cortical regions as illustrated in Table 4. There were also a number of regions that were lateralized to either the left or to the right during PIT, also presented in Table 4. Sub-cortical regions with greater BOLD signal differences in patients compared with controls during the task included bilateral putamen, caudate, thalamus, brainstem and right accumbens. Fig 2 presents the imaging results of patient group, the control group and the difference between patients and controls when engaged in the PIT task. Fig 3 is the graphical representation of percentage signal change of the regions illustrated in Fig 2.

**Table 4:** Group differences for PIT task during second level analysis.

	Co-ordinates			z-stat	Cluster size
	x	y	z		
ACC (L)	-8	30	22	3.09	1
ACC (R)	12	36	16	2.70	1
Accumbens (R)	10	12	-8	2.83	1
Amygdala (L)	-32	-6	-24	2.76	1
Angular gyrus (L)	-44	-56	50	4.53	2
Caudate (L)	-10	14	0	3.78	1
Caudate (R)	14	16	0	3.86	1
Cuneus (L)	-8	-80	34	3.36	3
Cuneus (R)	8	-78	34	2.91	3
Frontal orbital cortex (L)	-28	20	-12	2.46	1
Frontal orbital cortex (R)	40	20	-8	2.79	1
Frontal pole (L)	-30	58	14	3.39	1
Inferior frontal pars opercularis (R)	56	18	0	3.04	1
Inferior frontal pars temporalis (R)	52	24	2	3.45	1
Insula cortex (L)	-30	20	-4	2.76	1
Insula cortex (R)	36	18	-2	2.56	1
Middle frontal gyrus (L)	-44	18	38	3.67	1
Middle frontal gyrus (R)	48	20	40	2.46	1
Pallidum (L)	-12	4	2	3.27	1
Paracingulate cortex (L)	-4	32	34	4.02	1
Paracingulate cortex (R)	4	32	34	2.88	1
Parahippocampus posterior (L)	-22	-24	-28	2.57	1
Parietal operculum (L)	-60	-30	20	2.96	2
PCC (L)	-4	-50	32	3.33	3
PCC (R)	6	-46	32	3.51	3
Precuneus (L)	-4	-74	42	3.43	3
Precuneus (R)	2	-74	42	3.88	3
Putamen (L)	-24	6	-2	3.13	1
Putamen (R)	22	10	-2	3.17	1
SI(L)	-58	-22	26	3.13	2
Substantia nigra/ventral tegmental (L)	-4	-20	-14	3.19	1
Superior frontal gyrus (L)	-2	32	46	2.93	1
Superior frontal gyrus (R)	22	22	46	3.27	1
Superior parietal cortex (L)	-40	-48	50	4.09	2
Superior temporal gyrus posterior (L)	-58	-32	0	3.04	2
Supplementary motor cortex (L)	-4	0	50	3.09	1
Supramarginal gyrus, anterior division (L)	-52	-32	42	3.66	2
Supramarginal gyrus, posterior division (L)	-52	-48	42	4.17	2
Thalamus (L)	12	-12	6	3.37	1
Thalamus (R)	-12	-20	6	4.00	1

Anatomical locations and peak activation co-ordinates (in MNI 152 space) extracted from brain regions that were found to be significantly different between patients and controls (patients > controls) at Z>2.3 and cluster corrected *p<*0.05. Z stat refers to the peak voxel. The z-stat comes from the corrected clusters, the z-stats themselves are not corrected Anatomical locations clustered in 3 areas: cluster 1–13219 voxels, cluster 2–3063 voxels and cluster 3–3176 voxels with a resolution of 2mm x 2mm x 2mm.

In the supplementary second level analysis, BOLD responses during the picture task were positively correlated with higher anxiety scores and higher catastrophising scores across the group as a whole and these regions are illustrated in Table 5. Patients reported the higher anxiety and catastrophising scores compared to controls who did not report task related anxiety or catastrophising. Anatomical locations and peak activation co-ordinates were extracted from brain regions that were found to correspond significantly with higher anxiety and catastrophizing scores at Z>2.3 and these were cluster corrected *p<*0.05. Z stat refers to the peak voxel. The z-stat comes from the corrected clusters, the z-stats themselves are not corrected. The anxiety related anatomical locations were all in one cluster with a size of 2409 voxels with a resolution of 2mm x 2mm x 2mm. The catastrophizing related anatomical locations were in four clusters, cluster 1 689 voxels, cluster 2 709 voxels, cluster 3 748 voxels and cluster 4 904 voxel with a resolution of 2mm x 2mm x 2mm.

**Table 5:** BOLD regions correlating with higher anxiety and catastrophizing scores.

	Co-ordinates			z-stat	Cluster
	x	y	z		
*Regions corresponding to higher anxiety scores*					
Cuneal cortex (L)	-6	-88	30	3.41	1
Cuneal cortex (R)	10	-84	24	2.66	1
Intracalcarine (R)	6	-78	4	2.48	1
Lateral occipital cortex (R)	28	-84	36	2.50	1
Lingual gyrus (L)	-16	-78	-2	2.84	1
Lingual gyrus (R)	8	-70	-2	3.11	1
Occipital fusiform (L)	-20	-80	-2	3.44	1
Occipital pole (R)	6	-94	6	3.04	1
Precuneus (L)	-6	-60	36	3.84	1
Precuneus (R)	6	-74	38	3.08	1
Temporal occipital fusiform (L)	-26	-58	-8	2.80	1
*Regions corresponding to higher catastrophizing scores*					
Inferior temporal gyrus (R)	54	-52	-14	2.87	1
Lateral occipital cortex (R)	50	-62	18	2.53	3
Lingual gyrus (R)	8	-58	-4	2.46	3
Middle temporal gyrus (R)	66	-42	-8	3.17	1
Precuneus (R)	30	-58	10	2.89	3
Temporal occipital fusiform (R)	38	-42	-14	2.88	1
Cerebellum:					
Crus II (R)	22	-76	-38	2.68	2
V (R)	8	-54	-6	2.95	3
V IIb(R)	30	-68	-54	2.50	2
VIIIb (R)	12	-48	-58	3.35	4
Crus II (L)	-24	-74	-44	2.41	4
IX (L)	-12	-48	-50	2.99	4
VIIIa (L)	-22	-60	-54	2.90	4
VIIIb (L)	-14	-42	-52	3.33	4
X (L)	-18	-38	-44	2.47	4
Brain stem	-2	-44	-56	3.19	4

The accompanying analysis using TSK scores did not reveal any BOLD signal correlations across the group in either comparison (patients > controls; controls > patients).

## Discussion

The PIT was developed, using pictures from PHODA, to investigate BOLD signal changes in patients compared to controls when viewing pictures of daily activities. We found that a number of pain processing regions including the insula, ACC and inferior and superior parietal cortices were seen to have increase BOLD responses in patients compared to controls in response to observing the photographs and rating the perceived level of anxiety at performing the activity. Similarly, increased BOLD responses in patients compared to controls in the frontal pole, paracingulate and the supplementary motor cortex may be suggestive of a memory component to the responses. The left amygdala, bilaterally the insula and the orbitofrontal cortex, substantia nigra/ventral tegmentum, putamen, thalamus, pallidum, inferior parietal (supramarginal and angular gyrus) and cingulate cortex were also seen to have differences in BOLD signal changes in patients compared with controls and many of these regions are also associated with general phobic responses [57]. Therefore, we suggest that PIT is a useful task to explore pain- and movement-related fear and anxiety in fMRI studies.

A previous study by Shimo et al [60] in their visualization of painful experiences in low back pain study supported the usefulness of picture tasks to explore pain- and movement-related anxiety and fear in chronic pain populations. In common with our study, a number of regions of activity were found in patients but not controls including the insular cortex and the supplementary motor cortex. Shimo et al [60] concluded that activation in these areas might be associated with preparation for protective behavior against pain. They also found increased activation in the cerebellum, a region we found to correspond with higher catastrophizing across the whole group and where patients but not controls rated the higher scores. Both studies saw activation in the patient groups in the PCC, a region described by Shimo et al to possibly reflect the negative emotion and the pathologic state of pain. It is also conceivable, as discussed below that activation in the PCC was also part of abnormal Default Mode Network activity. The fusiform gyrus and the angular gyrus regions were also seen to be activated in patients and not controls and this was proposed by Shimo et al to be associated with facial recognition and empathy respectively and may result from visual recognition of the stimuli leading to imagined self pain or empathy towards the individual in pain in the picture.

The movement-related anxiety and fear associated with PIT, we believe, is part of a process that involves regions involved in the affective-motivational dimension of pain which appraises the value of the stimuli and motivates subsequent behavior and regions associated with anxiety. Regions involved in the affective-motivational dimension of pain were observed in patients compared with controls in the initial second level analysis when undertaking PIT and they include the insula cortex and rostral ventral ACC [78], inferior and superior parietal cortices and thalamus [79–82]. The precuneus, in combination with the cuneal cortex, also has a role in attentional biases [83] and has a role in attentional orientation [84] and enhancing attention for the processing of threatening events [85]. PIT was not designed to evoke unpleasantness; the participants in the pictures demonstrate neutral faces and do not exhibit pain behavior. Our results suggest that the thought of undertaking the activities seen by the patients may cause unpleasantness as brain regions involved in the affective-motivational domain of pain are implicated.

Regions involved in the cognitive-evaluative dimension, also saw increased BOLD responses in patients compared with controls during the PIT task. It has been shown that when individuals view pictures of body parts in painful situations, regions such as the inferior parietal cortex and ventral premotor areas are activated [86]. The joint activation of inferior parietal and inferior frontal cortex appears to be a key feature of action observation [87]. The recruitment of this network relates to predicting and understanding the outcome of the shown situation and we propose that patients process the pictures differently than controls because of differing perceptions of the outcome; patients but not controls are likely to consider pain as the outcome of undertaking the tasks in PIT. This is reinforced by the picture perceived pain ratings provided by participants following the scanning session. Patients but not controls perceived that the activity pictures would result in moderate to severe pain should the activity presented be undertaken.

Higher anxiety ratings and catastrophizing scores correlated to BOLD activation in the lateral occipital cortex, precuneus, lingual gyrus and temporal occipital fusiform gyrus. Anxiety ratings also showed increased BOLD activation in the occipital pole, intracalcarine cortex, and cuneal cortex and higher catastrophising scores in the inferior and middle temporal gyrus, multiple regions in the cerebellum and brain stem. Since both catastrophizing and diffuse inhibitory noxious control are involved in pain processing, they may be associated with each other. Evidence does exist to support this based on connectivity between brain areas associated with catastrophizing [88, 89] and the brain stem [90–93] associated with the diffuse noxious inhibitory control effect [94]. Catastrophizing may be affecting the sensitivity to pain through indirectly acting via the inhibitory pathways. The catastrophizing cortical regions were all lateralized to the right, consistent with previous research in anxious arousal [95–97]. Anxious arousal is characteristic of high-stress situations that are impending rather than in the distant future [97]. Therefore, anxious arousal may explain the lateralization of the catastrophizing regions.

The occipital and calcarine regions of the visual cortex can be modulated by emotional visual stimuli [98, 99] with increased fusiform activation [98, 99] and increased amygdala activation [99] facilitated by the saliency of the images shown. The cuneus, also involved in visual processing has also shown increased activation for affective stimuli [100]. The lingual gyrus has been implicated in visual memory [101]. It has been postulated that the mechanisms for increased activation in these regions is that projections from the limbic circuitry enhance activation in the ventral stream when viewing emotional content and affective salience of a stimulus and memory directly influences visual activity [102]. Similarly occipital poles are modulated by salient, emotional visual stimuli [103] possibly via back projections from the amygdala [104].

Comparison of patients and controls when undertaking PIT illustrated increased BOLD activity in patients in a number of regions involved in memory processing such as the frontal pole [105], paracingulate (reality monitoring in relation to memory processing) [106] and the supplementary motor cortex (important for tasks that demand retrieval of motor memory and for motor planning) [107]. We proposed that PIT was salient to patients given the BOLD responses in the memory centers which may indicate that patients were imagining the movement in the tasks and remembering pain associated with them.

In their quantitative meta-analysis, Etkin and Wager [57] revealed consistent amygdala hyperactivity in anxiety disorder and in fear conditioning in healthy subjects. They concluded that amygdala hyperactivation reflects a common exaggerated engagement of fear circuitry. The amygdala is critical for learning the aversive properties of events through fear conditioning, across species and stimulus types [55, 57, 108–112], the left amygdala being more likely to have increased activity when participants are perceiving instances of fear [55, 110, 113–115] than when perceiving or experiencing any other emotion categories [113].

The insula is strongly interconnected with the amygdala, hypothalamus and periaqueductal gray (PAG) [116] regions involved in a ‘pathway to fear’ [117]. The insula also regulates the autonomic nervous system [118] and is activated during the processing of a variety of negative emotions [119]. Thus it is notable that insula hyperactivity was consistently observed in all anxiety disorders reviewed by Etkin and Wager [57] as well as during normal fear conditioning [113]. Insula hyperactivity is therefore likely to reflect increased activation of a network responsible for generating fear responses to symptom provoking stimuli [57]. Though the insula has received less intense study than the amygdala in the context of negative emotional processing, its important role is suggested by its more frequent association with activation in the amygdala than with activation in other cortical regions, thereby suggesting a high degree of functional similarity to the amygdala [103]. Although both the insula and amygdala had increased BOLD activity in patients compared to controls when viewing pictures and rating perceived anxiety, there were no correlations when the actual anxiety scores were regressed into the model. This is interesting as this may support the contention that movement-related anxiety is largely an automatic emotional response that is not well identified using self-report measures.

The anterior mid cingulate cortex (aMCC) demonstrated increased BOLD signal changes in patients compared to controls, lateralised to the left, during PIT (x -2, y 16, z 22; zstat 2.88). This region is part of an intrinsic brain network that shows increased activity when stimuli in the environment are personally salient [120]. The aMCC has been shown to have consistent increases in activation during instances of fear perception than the perception of any other emotion category [113].

Our patients rated themselves as low kinesiophobics on the TSK, scoring well below the cut-off point between low and high kinesiophobics at a score of 37 [23]. This was despite high self-reported anxiety scores. This is not unexpected, given that the TSK has been shown to lack sensitivity as a tool for measuring pain related fear of movement [32, 121] and this may account for, in part, the contradictory findings in pain-related anxiety and fear-avoidance studies. Patients may harbor undisclosed or unrecognized anxiety related to movement, and movements that provoke anxiety are not defined in the TSK, therefore this tool may prove unsuitable in tapping into specific individual fears [32]. The pictures presented to participants allowed them to view specific activities and imagine their anxiety in relation to undertaking them, unlike TSK where beliefs about movement and exercise in general are used to rate the level of anxiety and movement-related fear. The measurement of movement-related anxiety and fear is complicated by the problem that the individual may not be consciously aware of how they acquired these anxieties and fears and may not be consciously aware of them [122]. This means that self-report measures could underestimate anxiety and pain- and movement- related fear in those that are not consciously aware of what provokes the anxiety [32].

Past reviews have suggested strong support for the Fear-Avoidance (FA) Model [20, 30, 123] and it has widely influenced clinical practice despite methodological gaps in its supporting research. Much of the supporting literature has been based on correlational analyses [124], and of the limited prospective studies conducted, several do not lend support for the model’s core predictions [125]. Future research aiming to test model predictions will need to use innovative designs [126]; integration of biological factors with current FA variables has been suggested as a consideration for future refinement [124]. As chronic pain develops several biological changes occur (e.g. central sensitization, inflammatory response, brain function and DMN activity) and links between these biological changes and pain-related anxiety and fear as illustrated in the FA Model have been suggested [89, 127, 128].

Changes in Default Mode Network (DMN) activity appear to occur as a result of chronic pain. The DMN is the network that decreases its activation during a task compared to the average brain activity at rest and includes the precuneus, PCC and medial frontal cortex [48, 129, 130]. Recently, the angular gyrus has also been implicated in the DMN [131]. In the present study, regions in the DMN including the angular gyrus, remained active or were less deactivated during the task in patients with CMSKP compared to healthy controls. This is consistent with a number of studies [48, 130, 132] where it appears that long term pain affects brain function; the brain is never truly at rest because it is constantly processing pain.

### Limitations

Patients continued with their routine medications and no new drugs were commenced three months prior to the study. There are problems in studying patients with severe and complex chronic pain, such as seen in those referred to specialist pain centers. Problems exist in extricating pain-related effects from those resulting in pain treatments, especially opioids [133]. Therefore, it has been suggested that a pragmatic approach to studying this group of patients is required [133]. It was inappropriate to ask patients to stop their drug regimens from a clinical perspective. Patients were not asked to stop their medications and therefore, the functional and structural changes as a result of taking these drugs over a long period [134] may have an impact on results. However, all patients had stable treatment regimens that had not been altered during the 3 months prior to imaging.

Given the age ranges and the known interactions between age and brain structure in chronic pain, there may be an age by group interaction that would add variability to the data but the sample size was too small to test such an interaction. The heterogeneity in age, pain sites and duration of pain across patients does adds variability to the study. However, the impact of the heterogeneity is that statistical significance is harder to achieve and had the patient group been more homogenous, our results may have been more significant.

## Conclusion

The Picture and Imagination Task (PIT), largely developed from the Photographs of Daily Activity assessment tool [62] was designed for a CMSKP population in whom pain is naturally occurring and neuroimaging research is relatively sparse. Through the use of our paradigm we demonstrated BOLD signal differences between those with CMSKP and controls. The results were not due to the presence of ongoing pain while patients were in the scanner; the pictures did not provoke physical pain and participants had previously reported that lying down did not provoke spontaneous pain. PIT may help as a tool in neuroimaging research to study integration of the factors that influence disability with movement-related fear and anxiety. PIT identified a difference in neural activity in patients compared with controls to the mere thought of undertaking an activity. This has important implications for how pain and movement-related anxiety and fear are assessed and managed.
